# Time for a renewed focus on the role of cleaners in achieving safe health care in low- and middle-income countries

**DOI:** 10.1186/s13756-021-00922-x

**Published:** 2021-03-24

**Authors:** Julie Storr, Claire Kilpatrick, Karen Lee

**Affiliations:** 1 KS Healthcare Consulting Ltd, Glasgow, UK; 2 Glasgow Caledonian University, Glasgow, UK

**Keywords:** Environmental cleaning, Environmental hygiene, Antimicrobial resistance, Water, Sanitation, Hygiene, Infection prevention, Cleaners, COVID-19

## Abstract

Water, sanitation and hygiene, collectively known as WASH, is an enabler of infection prevention and control (IPC), both of which contribute to safe, quality health care and the prevention of spread of antimicrobial resistance (AMR). This discussion paper considers the importance placed on the role of hygiene, including cleaning and those who clean, in health care facilities, within the context of existing data, guidelines and initiatives. Informed by this, the paper presents five areas for consideration that have the potential to strengthen and further demonstrate the value of this important cadre of staff and their role in clean, safe healthcare, particularly in low- and middle-income countries. The considerations centre around actions to overcome the current data gaps, including the paucity of national data on environmental cleaning and the training of cleaners; strengthening the implementation of norms and standards; combining global and national advocacy efforts; revisiting investment; and addressing research gaps on the issue. The need to act, in line with WHO and UNICEF recommendations to address this overlooked and undercompensated workforce and to elevate their status as important contributors to IPC, WASH and AMR is a pressing one.

## Background and introduction

Water, sanitation and hygiene, collectively known as WASH, is an enabler of infection prevention and control (IPC), both of which contribute to safe, quality health care and the prevention of spread of antimicrobial resistance (AMR) [1]. Considering the component parts of WASH, without access to water a hospital cannot support a safe, hygienic environment or deliver clean care. In addition to being required for drinking, clinical procedures and activities, laundry and cooking, water is needed for toilets to function (sanitation), and cleaning, both environmental and of hands (hygiene) [2]. WASH therefore is part of the supportive environment that enables cleaning staff, as one part of the workforce, to fulfil their role. Within the context of IPC, the World Health Organization (WHO) describe cleaning staff as an important part of the workforce and highlight this group as a target for training, given their role in overall health service delivery [3].

This discussion paper considers current information that places importance on the role of hygiene, including cleaning, and of those who clean in health care facilities. It does so within the context of existing data, guidelines and initiatives. It also outlines those who clean as a central and necessary part of the health workforce, drawing on some of the themes emerging from recent studies. Informed by this, five areas for consideration are outlined in the conclusion in order to strengthen and further demonstrate the value of this important cadre of staff and their role in clean, safe healthcare, particularly in low- and middle-income countries (LMICs).

## Data on the extent of the problem

The lack of basic WASH in health facilities has been highlighted as a major issue in LMICs, particularly across Africa, and some countries in south east Asia and the eastern Mediterranean region [4] and efforts are underway to address this. Data from countries representing nearly 60% of the population of all LMICs found that most healthcare facilities in these settings have inadequate environmental conditions i.e. WASH, and insufficient availability of resources necessary for standard IPC precautions [5] which includes environmental cleaning. In 2020 WHO and UNICEF published both the results of their periodic assessment of the global state of WASH in healthcare facilities and proposed actions for addressing improvement, building upon recent international calls to action, commitments and resolutions [6]. It is important to note that the approach to data collection in this report used available national data to generate internationally comparable estimates for a core set of standardized indicators covering WASH, waste management and environmental cleaning. The unsafe state of WASH in health care facilities in LMICs is clear, with the problem particularly profound in the 47 least developed countries (LDCs) represented. Specifically, one in four health care facilities lack basic water services, and one in 10 have no sanitation services. Across the 47 LDCs, half of health care facilities lack basic water services. The findings can be interpreted as follows; globally 1.8 billion people use health care facilities that lack basic water services and 800 million use facilities with no toilets. There remain, however, major data gaps on the state of environmental cleaning in health care, with only 12 countries having sufficient data on the availability of cleaning protocols and the availability of training for those with cleaning responsibilities. This is a reality check for environmental cleanliness opportunities and the conditions and challenges faced by those who both clean and require clean healthcare facilities in LMICs.

## Related norms, standards and initiatives

WHO and UNICEF have developed numerous documents, including guidelines, norms and standards, issued calls to action and launched initiatives that directly or indirectly relate to those who clean health care facilities. These have been supported by many development partners, non-governmental organisations, academic institutions and professional bodies. In what is considered to be the definitive standard addressing environmental hygiene and health, including cleaning, WHO stated that cleaning staff should be made aware of the importance of their role and should be able to apply the basic principles of IPC to their daily work, supported by appropriate training and management [7].

WHO’s comprehensive IPC guidelines and related minimum requirements for the national and facility level [3, 8–10], implementation guidance on a specific resistant organism of concern [11] and core competencies for IPC professionals [12] all highlight a number of factors related to cleaning specifically. Box 1 lists these and related initiatives.

**Box 1:** Summary of related guidance and initiatives

Guidance and standards
WHO (2008) Essential Environmental Health Standards in Health Care
WHO (2016) Guidelines on core components of infection prevention and control programmes at the national and acute health care facility level
WHO (2019) Minimum requirements for infection prevention and control. Geneva: World Health Organization
CDC and ICAN (2019) Best Practices for Environmental Cleaning in Healthcare Facilities in Resource-Limited Settings. Atlanta, GA: US Department of Health and Human Services
WHO (2020) Cleaning and disinfection of environmental surfaces in the context of COVID-19. Interim guidance
WHO (2020) Core competencies for infection prevention and control professionals. Geneva: World Health Organization
World Health Assembly Resolutions
World Health Assembly (WHA 72). 2019. Agenda Item 6.6, Patient Safety. Resolution on Water, Sanitation and Hygiene in Health Care Facilities
World Health Assembly (WHA 73). 2020. Agenda Item 3. Resolution on COVID-19 Response
Calls to action/initiatives
WHO (2015) Global Action Plan on Antimicrobial Resistance
WHO/UNICEF (2018) Meeting the challenge: responding to the global call to action on WASH in health care facilities
WHO (2020) Urgent health challenges for the next decade
WHO Patient Safety Flagship: A Decade of Patient Safety 2020–2030
WHO (2021) Global Patient Safety Action Plan 2021–2030. Towards Eliminating Avoidable Harm in Health Care. Third draft, January 202
Implementation resources
WHO (2017) Water and Sanitation for Health Facility Improvement Tool (WASH FIT): a practical guide for improving quality of care through water, sanitation and hygiene in healthcare facilities
WHO, UNICEF (2019) Water, sanitation and hygiene in health care facilities: practical steps to achieve universal access
WHO (2019) Implementation manual to prevent and control the spread of carbapenem-resistant organisms at the national and health care facility level. Geneva: World Health Organization

The global IPC guideline recommendations of 2016, referred to as the ‘Core Components’ [3] and more recent Minimum Requirements for IPC programmes [10] state that patient care activities should be undertaken in a clean and/or hygienic environment that facilitates practices related to the prevention and control of healthcare associated infections (HAI), as well as AMR, including all elements around WASH infrastructure and services and the availability of appropriate IPC materials and equipment. They further emphasise that cleaners and janitorial staff should be trained on specific aspects of IPC. In fact, cleaners are identified as key personnel that support health service delivery and singled out as a target for IPC training. WHO publications on the practical steps and improvement approaches targeted specifically at WASH in health care facilities, echo this [13, 14].

In 2019, a novel best practice guidance focused on environmental cleaning in healthcare facilities in resource-limited settings was issued by the Centers for Disease Control and Prevention (CDC) and Infection Control Africa Network (ICAN) [15], with support from WHO. The document outlines five elements that are needed for effective environmental cleaning programs, regardless of the type of facility (Box 2). These five elements are interlinked with no hierarchy of importance and reinforce the need for a comprehensive, multifaceted approach, that addresses not only training but also infrastructure, policies and measurement as well as organizational factors including leadership and culture.

**Box 2:** Implementing environmental cleaning programs: the five key programme elements [15]

1. Organization/administration
2. Staffing and training
3. Infrastructure and supplies
4. Policies and procedures
5. Monitoring, feedback and audit

Objective three of the global action plan (GAP) on AMR [16] also focuses on the need for effective hygiene, together with sanitation and IPC measures, in order to reduce the incidence of HAI and to support global efforts to tackle AMR. The GAP highlighted the need for awareness, education and training on hygiene including WASH and IPC, which is in line with the WHO IPC and WASH recommendations for training. It calls for action by international and national partners to help build an investment case for improved hygiene. In 2018, the United Nation’s Secretary General also issued a call to action on WASH in health care facilities [17] which garnered global support for translating this into action [18, 19]. Importantly, in January 2020, at the start of the COVID-19 pandemic, WHO identified *keeping healthcare clean* as one of thirteen urgent health challenges [20]. The pandemic has further stimulated international guidance on cleaning and disinfection of environmental surfaces [21] shining a light on the issue. Although designed for a specific microorganism, the guidance is clear in its recommendation that training of cleaning staff should be based on local protocols and national guidelines. The guidance states that training should be structured, targeted, and delivered in the right style (e.g. participatory, at the appropriate literacy level), and it should be mandatory during staff induction.

Finally, as the world embarks on a decade of patient safety [22] a new global action plan addressing patient safety is under development [23]. Strategic objective 3: “Assure the safety of every clinical process”, emphasizes the need to ensure a clean and hygienic environment that incorporates a WASH infrastructure, with availability of appropriate IPC materials and equipment. The WHO and UNICEF data and progress report of 2020 [6] with its rallying call to put the ‘fundamentals first’, concluded with four recommendations, one of which stated that “these non-health care providing staff (those who clean), who are often overlooked and undercompensated, need to be recognized and elevated within health workforce policies, programming and budgeting”.

## A growing focus on those who clean within the literature

Within the academic literature, a focus on the training and empowerment of cleaning staff in LMICs has gathered pace in recent years. Notable in this arena is the work of the SOAPBOX Collaborative, an initiative now under the auspices of the London School of Hygiene and Tropical Medicine (LSHTM) [24]. A training package (TEACH CLEAN) and associated research publications on cleaning and those who clean in maternal and new-born settings in LMICs, was developed as part of the Collaborative. The associated publications that drove the development of this package focus on hygiene in maternity units [25, 26] and reveal limited formal training for ward cleaners, and a lack of written policies and protocols and poor monitoring and supervision. They also highlight the neglected role of cleaners and their low status within facilities, wider societal marginalisation, and poor pay and working conditions as contributory factors, leading to the lack of prioritisation placed on health facility environmental hygiene. One study described cleaners as an invisible workforce [26]. This work concluded that training for both healthcare providers and ward cleaners represents an important opportunity for quality improvement. Other studies have also highlighted deficiencies in the training, knowledge and practices of cleaning staff in LMICs [27, 28] and challenges relating to role extension and the blurring of boundaries between cleaning and caring [29, 30].

Although predominantly focused on high-income countries (HIC), a number of publications have highlighted the importance of a clean environment, including in the context of resistant organisms [31] and shed useful insights into the motivation of those who clean, with pay an important but not the sole motivation [32]. Current research by Mitchell and also Hall and colleagues [33, 34] is adding further valuable insights into the implementation of cleaning strategies, although again from a largely HIC perspective. The ‘Clean Hospitals’ initiative established by a group of healthcare professionals and corporate entities called for a re-evaluation of how hospitals view environmental hygiene, particularly a shift towards seeing cleaning as an investment that adds value, rather than a pure cost burden [35]. This initiative states that it is currently developing a training curriculum and training modules on hospital environmental cleaning. In the medium term, it plans to develop a self-assessment framework for hospitals to determine the state of environmental hygiene and drive improvement. Peters et al. recall how WHO’s first global patient safety challenge, Clean Care is Safer Care [36] focused on hand rather than environmental hygiene, and ponder whether employing a similar campaigning approach might be worth replicating for hospital cleaning. WHO’s global annual campaign is widely considered to have catapulted hand hygiene to the global stage and resulted in tangible awareness and degrees of improvement at the healthcare facility level.

The message from this literature is the ongoing neglect of cleaning and those who clean in health care that warrants further attention. The neglect is both in terms of the training and development of this often-overlooked cadre of the workforce and a general undervaluing of their role. Where IPC and other training is in place, inadequacies seem to exist and training is often sub-optimal or not employed within a multimodal, participatory approach resulting in a negative association between training and knowledge (and practice). A lack of written policies and protocols on environmental cleaning, coupled with sub-optimal monitoring and supervision is also a feature. An opportunity exists to engage and capacitate cleaning staff as part of the other initiatives already underway.

## Considerations for the future

Considering the current pandemic, the international guidance and initiatives and the emerging studies in this evolving field, it seems timely to further explore how best to demonstrate the value of those who clean and strengthen support for the enhancement of their role within existing initiatives. We present five areas for consideration going forward:

1. Enhancing the available data. Data on the extent of the WASH problem exists and has been described as shocking, but the global indicator on environmental cleaning remains data poor. Further work is required to understand the low reporting on cleaning and how countries can be stimulated to prioritise the collection of more and better data [6]. A key question is, can the WASH, IPC, AMR, and for example the maternal and child health communities come together to help address this data vacuum, given the powerful role of data in supporting advocacy and improvement?
2. Implementation of norms and standards. Norms and standards exist at the global level that address cleaning, and in part the role of cleaning staff, in the context of IPC, AMR and WASH and now COVID-19. Associated global implementation resources promote the importance of a facility culture that supports hospital cleaning staff. However, further work is needed to ensure successful dissemination of all the available information. In order to empower those who clean, greater awareness of the existence of these guidelines and tools is needed across all relevant sectors. To realise this the promotion of exemplars such as the TEACH CLEAN package should be taken, so that an approach to training those who clean can be adopted at scale. This is one specific way dissemination and therefore implementation can be supported.
3. Combining advocacy efforts. Resolutions, campaigns and calls to action exist and are ripe for further development. For example, combining hand hygiene initiatives with the need for action to maintain clean environments in order to complement the impact of hand hygiene action at the right times. The current melee of global campaigns that countries are called on to be involved in may be resulting in competition and dilution of messages, rather than being complementary. A meta-campaign that unites existing efforts and resources would be a novel and progressive move, driven from the global level. This will also support political advocacy required to strengthen the case for national data collection on the state of cleaning in health care facilities as well as the training of those who clean.
4. Revisiting investment. Action on and investment in cleaning and those who clean in LMICs is one way countries can respond to all global calls to action and resolutions. Greater integration and collaboration amongst organizations committed to IPC, WASH and AMR, including NGOs, would support this.
5. Addressing the research gap. Research and more broadly publications from LMICs are limited. This has also been noted by the IPC community [37]. However, where it exists it does highlight that cleaning staff are a neglected part of the health workforce and their training is either non-existent or sub-optimal. In terms of the research agenda, a critical question moving forward might be, how can findings from high income countries support or be combined with those on the neglected frontline in LMICs?

## Conclusion

Hygiene and cleanliness in healthcare (and beyond) is firmly in the international spotlight, not least because of COVID-19. As the world pursues an array of global initiatives and challenges related in some way to addressing the lot of those who clean health care facilities, united efforts by the IPC, WASH and AMR community at the very least, remains an ongoing need. COVID-19 could be a game changer for sustainable change in this area. It has reminded the world that action is needed to address the abysmal state of WASH and IPC in healthcare (including environmental hygiene and cleanliness) and because of the nature of pandemics, urgency has been injected into the proceedings. In an intra and post-COVID-19 world, more countries are likely to embrace or be compelled to embrace safer, better quality, more resilient healthcare services. Whatever world order emerges during the 2020s, it is clear that investment in those who make the world safer with every ‘sweep of the mop’ is critical. Those who clean contribute in numerous ways to the standard of hygiene in health care, and as such overall health outcomes. In light of this, the need to take action, in line with WHO and UNICEF recommendations to address this overlooked and undercompensated workforce and elevate their status as important contributors to IPC, WASH and AMR is a pressing one.

## Data Availability

Not applicable.

## Abbreviations

AMR: Antimicrobial resistance
CDC: Center for disease control
GAP: Global action plan
HAI: Health care-associated infection
HIC: High-income countries
ICAN: Infection control Africa network
IPC: Infection prevention and control
LSHTM: London School of hygiene and tropical medicine
LMIC: Low- and middle-income countries
UNICEF: United Nations International Children's Emergency Fund
WASH: Water, sanitation and hygiene
WASH FIT: WASH facility improvement tool
WHO: World Health Organization
